# A Comparative Study of Ischemia-Modified Albumin: A Promising Biomarker for Early Detection of Acute Coronary Syndrome (ACS)

**DOI:** 10.7759/cureus.44357

**Published:** 2023-08-30

**Authors:** Pranav Jawade, Kishor M Khillare, Sangram Mangudkar, Amit Palange, Jagannath Dhadwad, Madhura Deshmukh

**Affiliations:** 1 General Medicine, Dr. D.Y. Patil Medical College, Hospital and Research Centre, Pune, IND; 2 Central Research Facility, Dr. D.Y. Patil Medical College, Hospital and Research Centre, Pune, IND

**Keywords:** acs (acute coronary syndrome), cardiac diagnostic tool, cardiac troponin i, ecg, ischemia-modified albumin

## Abstract

Introduction

The second most common cause of emergency department (ED) visits is chest pain and discomfort. Timely identification or threat stratification is crucial for identifying high-risk individuals who benefit from sophisticated diagnostic investigations (including cardiac biomarkers) and early relevant therapies. We aimed to assess the levels of ischemia-modified albumin (IMA) and also to study its sensitivity and specificity in comparison with cardiac troponin T/troponin I and electrocardiogram (ECG) (alone and in combination) in the diagnosis of acute myocardial infarction.

Methods

Adults (either gender) presented at the ED of a tertiary care centre with classical chest pain suggestive of angina pectoris or angina-like chest pain and ECG changes suggestive of ACS, ST-elevation myocardial infarction (STEMI), non-ST elevation myocardial MI (NSTEMI), and unstable angina, within three hours of onset were enrolled. Demographic and clinical information was recorded. ECG, haematological investigations like complete blood count, blood sugar level, lipid profile, IMA, troponin I, and creatinine kinase-MB (CK-MB), and radiological investigations like 2D-echocardiography (2D-ECHO) and coronary angiography were performed.

Results

A total of 100 subjects were enrolled in the study out of which 50 were cases and 50 were controls. Cases were older as compared to controls (mean age 60.5 versus 46.0 years). Of the 50 cases, 33 (66%) were males. There were equal numbers of males (33 each) and females (17 each) subjects in both the groups. Typical chest pain, risk factors, and history of coronary artery disease (CAD) were higher in cases. ECG diagnosis revealed the presence of STEMI (52%) and coronary angiography revealed the presence of double vessel CAD (60%) in cases. Among controls, gastroesophageal reflux disorder was the most common cause of chest pain followed by costochondritis and pneumonia. Glucose (fasting and postprandial), all lipid profile parameters (except high-density lipoprotein) and IMA values were significantly higher in cases as compared to controls.

A combination of ECG+IMA has the highest sensitivity (90%) with 79% PPV in the diagnosis of ACS within three hours of the onset of chest pain, and ECG+IMA+2D-ECHO had similar results. However, ECG is equally sensitive. IMA alone has 64% sensitivity with 82% diagnostic accuracy which was higher than other biomarkers (CK-MB, cardiac troponin I).

Conclusions

As found in our study, among the biomarkers used, the diagnostic accuracy of IMA was the highest and better than that of cardiac troponin I and CK-MB. Although ECG is the preferred diagnostic tool for diagnosing ACS (STEMI, NSTEMI, and unstable angina) in patients presenting within three hours of the onset of chest pain, a confirmation can be done with the help of other diagnostic tests and investigations like serum IMA levels and further treatment can be initiated.

## Introduction

The second most common cause of emergency department (ED) visits is chest discomfort. Chest pain patients have the greatest variability in hospital admission rates, yet there are no obvious differences in the observed death [1]. The typical response to patients with non-specific breathing difficulties in the ED emphasised hospitalisation for further evaluation and risk stratification. This strategy was largely motivated by the concern of missing myocardial infarctions [2]. Due to the advancement of more complex cardiac biomarkers and the use of clinical summative assessments, clinicians have started to recognise a significant fraction of patients who may be securely evaluated in the ED and sent home. ED doctors prioritise assessing for potentially serious reasons when a patient enters with chest pain like pulmonary thromboembolism with or without deep vein thrombosis, acute coronary syndromes (ACS), aortic dissection etc.

Cardiomyopathy and hypotension are two clinical manifestations of ACS, encompassing various clinical manifestations. It is primarily brought on by the rupture plaques of atherosclerosis in coronary arteries, followed by thrombosis. This may result in a partial or complete obstruction of the coronary artery blood flow [3]. If the coronary artery occlusion persists and cardiac cell necrosis occurs, then the eventual result will be myocardial ischemia. This includes ST-elevation myocardial infarction (STEMI) and non-ST elevation myocardial infarction (NSTEMI). Despite improved awareness and diagnostic advancements, traditional approaches misdiagnose 2-8% of ACS patients. ACS patients are incorrectly sent home, resulting in an increased mortality rate, whilst non-ACS patients are taken to hospitals, straining hospital resources.

Timely identification or threat stratification is crucial for identifying high-risk individuals who benefit from sophisticated diagnostic investigations and early relevant therapies. The clinical presentation, physical examination, electrocardiogram (ECG) alterations, and testing of cardiac biomarkers of myocardial necrosis like cardiac troponin i and T are used to make the first assessment of patients with chest pain. Creatine kinase MB (CK-MB) is a cardiac biomarker that substitutes troponin I and T at times. It is similar to troponin and is released after cardiomyocyte necrosis. CK-MB is linked with infarct size and is inversely associated with long-term survival rates [4].

Recent advances in the understanding of ischemia have shown that it alters the albumin composition. Ischemia-related modifications, including hypoxia and metabolic acidosis, degrade the metal binding to the last amino terminal, resulting in the formation of ischemia-modified albumin (IMA). In 1990, IMA was first investigated as a diagnostic biomarker for myocardial ischemia patients. It is increasingly more widely acknowledged in therapeutic settings [5]. Albumin's metal-binding capacities are altered during ischemic events, thereby forming IMA which is a newer cardiac biomarker.

Here, in this study, we aimed to assess the levels of IMA in patients presenting with ACS and also to study the sensitivity and specificity of IMA in comparison with cardiac troponin T/troponin I and ECG (alone and in combination) as well as 2D-ECHO in the diagnosis of acute myocardial infarction.

## Materials and methods

This observational study was conducted at Dr. D.Y. Patil Medical College, Hospital and Research Centre, Pune, from October 2020 to November 2022. Adult patients (either gender) presented in the ED were screened with predefined inclusion and exclusion criteria. 

Those with classical chest pain suggestive of angina pectoris or angina-like chest pain, ECG changes suggestive of ACS (STEMI, NSTEMI, and Unstable Angina) and within three hours of the onset of symptoms, before any heparin on thrombolytic therapy initiated and willing to participate in the study, were included as ‘Cases’. We also enrolled patients with chest pain (angina-like) with musculoskeletal, GI, or other than cardiovascular causes as ‘Controls’ for comparative analysis. We excluded pregnant women and those patients who had signs and symptoms of acute mesenteric ischemia, clinically diagnosed acute renal failure/end-stage renal disease, peripheral vascular disease, brain ischemia, hypoalbuminemia, and cancer.

The study was approved by the Institutional Ethics Committee (Ref. No: IESC/PGS/220/19) and patients were enrolled after they gave written informed consent.

General, demographic, and clinical information was collected in a pretested proforma. Blood samples were collected in the proper vacutainers (ethylenediaminetetraacetic acid (EDTA), citrate, and serum separator vacutainers) and delivered to the Central Clinical Laboratory (CCL) of the hospital and the following investigations were performed using the listed instruments.

An ischemia-modified albumin assay kit (kit Manufacturer: Gcell) was used. The method employed was albumin-cobalt binding and the analyzer was Abbott Architect Ci 8200 Cat. NO: ETGS271X (Package size: R1: 1 x 45 ml, R2: 1 x 15 ml; Abbott, Illinois, USA)

Haematological and biochemical investigations

Haematological and biochemical investigations performed were CBC (haemoglobin, TLC, platelet count, MCV) using Beckman Coulter DxH 800 (Beckman Coulter, Brea, USA), fasting lipid profile (serum cholesterol, triglycerides, HDL, VLDL, LDL), troponin I, and blood sugar level (fasting, postprandial) using Architect c8000 equipment (Abbott, Illinois, USA), glycosylated haemoglobin A1c (HbA1c) using Bio-Rad D-10 instrument (Bio-Rad Laboratories), and CK-MB: Dimensions EXL 200 Integrated Chemistry System (Siemens healthineers, Erlangen, Germany). 

Radiological investigations

Radiological investigations performed were 2D-echocardiography (Philips EPIQ 7C, Philips, Amsterdam, Netherlands) and percutaneous coronary interventions (angiography/angioplasty) (Philips Allura Centron, Philips, Amsterdam, Netherlands).

Diagnostic criteria

The diagnosis was determined after carefully examining the clinical picture and pertinent investigations. Positive ECGs had two or more contiguous leads with ST elevations or depression >0.1mv or T wave inversion >0.2mv. Negative ECGs included those that are ambiguous or unexpected, such as left bundle branch block paced rhythm and sustained ST elevation after acute myocardial infarction. A positive angiogram was defined as stenosis >70% diameter reduction in any major epicardial vessel. Cardiac troponin I >10 ng/ml was considered positive. IMA values > 78.1 U/ Ml were considered positive for cardiac ischemia. There is no gold standard for myocardial ischemia, so IMA results were compared with the ‘FINAL HOSPITAL DIAGNOSIS’, representing the ‘GOLD STANDARD’ for the study. 

Statistical methods

Statistical analysis was conducted using Microsoft Excel (Microsoft 365, Microsoft Corporation, Redmond, USA) and IBM SPSS Statistics for Windows, Version 27 (Released 2020; IBM Corp., Armonk, New York, United States). Quantitative data were presented as median (IQR) since the data were skewed. Normality was checked using the Kolmogorov-Smirnov test. As the data were non-normal and data distribution was widely spread, the Mann-Whitney U test was performed for comparison between case and control groups. For all the tests, a p-value of <0.05 (two-tailed) was considered statistically significant. 

## Results

A total of 100 patients (50 cases and 50 controls) were enrolled in the study. Cases were older as compared to controls (mean age 60 vs.46 years). Of the total 100 patients, 66% were males and there were an equal number of male (33 each) and female (17 each) participants in both groups. A higher percentage of cases had typical chest pain as compared to controls (76% vs 28%). Seven (14%) of the cases and only five (10%) patients from the control group had a history of coronary artery disease (CAD). The most frequently observed risk factor among cases and control was hypertension (66% and 46% respectively). All other risk factors were found to be associated more with cases than the control group (Table 1). 

**Table 1:** General characteristics of the patients (n=100) Values displayed are median (min-max) for age and categorical variables are expressed as N (%)

	Cases (n=50)	Controls (n=50)
Baseline Characteristics		
Age (years)	60.5 (36 – 82)	46.0 (30 - 69)
Male (%)	33 (66%)	33 (66%)
Chest pain:		
Atypical	12 (24%)	36 (72%)
Typical	38 (76%)	14 (28%)
Risk factors:		
Hypertension	33 (66%)	23 (46%)
Diabetes	23 (46%)	8 (16%)
Smoking	22 (44%)	17 (34%)
Alcoholism	19 (38%)	11 (22%)
History of COVID-19	18 (36%)	7 (14%)
Obesity	17 (34%)	11 (22%)
Thyroid disorder	8 (16%)	9 (18%)
Others	10 (20%)	9 (18%)

ECG diagnosis revealed the presence of NSTEMI (44%) and STEMI (52%) in cases, while 76% of controls had normal ECG. Among cases, 70% had regional wall motion abnormalities (RWMA)+ and in controls 8% had RWMA+. Out of 50 cases, 48 cases underwent coronary angiography. The majority (60.4%) had double vessel disease (LAD+LCX was the most frequently affected vessel). 

Among cases (n=50), 32 (64%) had IMA positive, 15 (30%) had CK-MB positive, and 18 (36%) had Trop I positive results. All 50 controls also underwent IMA, CK-MB, and Trop I tests, but all three test results were negative. Among controls, gastroesophageal reflux disease (GERD) was the most common cause of chest pain followed by costochondritis and pneumonia. 

The IMA value was significantly higher in cases as compared to controls. (77.8 vs 21.1 U/MI). Cases also had significantly higher circulating glucose values (fasting, PP1, PP2) and HbA1C values. All lipid profile parameters, except HDL, were significantly higher in cases as compared to controls (Table 2).

**Table 2:** Biochemical parameters of cases and controls Values displayed are mean (±SD); Mann Whitney U test. p < 0.05; statistically significant* IMA: Ischemia-modified albumin, BSL-F: blood sugar level-fasting, BSL-PP1: blood sugar level postprandial 1, BSL-PP2: blood sugar level postprandial 2, HbA1c: glycosylated haemoglobin A1c, HDL: high-density lipoprotein, VLDL: very-low-density lipoprotein, LDL: low-density lipoprotein

Parameters	Cases	Controls	p-value
IMA U/Ml	77.8 (±17.2)	21.1 (±11.8)	<0.001*
BSL-F mg/dl	137.0 (±55.5)	100.9 (±12.6)	0.001*
BSL-PP1 mg/dl	208.0 (±84.7)	146.8 (±30.8)	<0.001*
BSL-PP2 mg/dl	208.3 (±74.3)	150.1 (±47.2)	<0.001*
HbA1c %	7.5 (±2.4)	6.0 (±0.7)	<0.001*
Total cholesterol mg/dl	177.7 (±29.6)	148.6 (±26.6)	<0.001*
Triglycerides mg/dl	146.9 (±53.8)	124.9 (±50.9)	<0.001*
HDL mg/dl	44.3 (±8.1)	44.5 (±6.1)	0.9
VLDL mg/dl	17.0 (±4.1)	13.2 (±3.3)	<0.001*
LDL mg/dl	116.2 (±26.7)	90.4 (±22.5)	<0.001*

Table 3 shows the ECG diagnosis of cases. Only 4% had normal ECG, 44% had NSTEMI, and 52% had STEMI on ECG.

**Table 3:** ECG diagnosis among cases (n=50) Data presented as n % STEMI: ST elevation myocardial infarction; NSTEMI: non-ST elevation myocardial infarction

Diagnosis	Frequency	Percent
STEMI	26	52
NSTEMI	22	44
Normal	2	4

**Figure 1:**
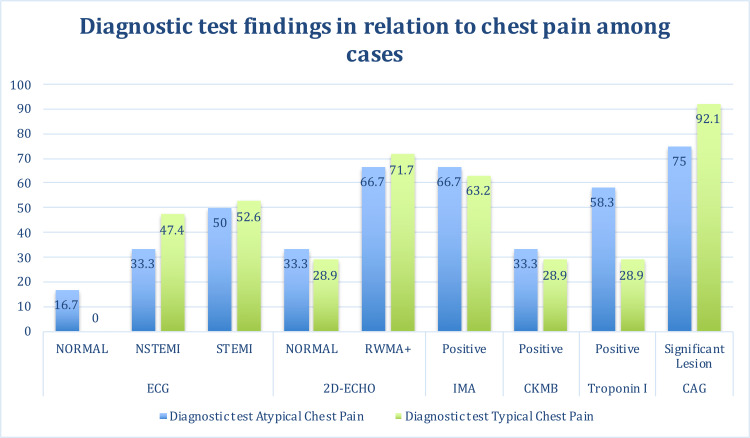
Diagnostic test findings in relation to chest pain (typical/ atypical) among cases (n=50) NSTEMI: Non-ST elevation myocardial infarction; STEMI: ST elevation myocardial infarction; RWMA: regional wall motion abnormality; ECG: electrocardiogram; 2D-ECHO: 2D- echocardiography; CKMB: creatine kinase MB; CAG: coronary angiogram

**Figure 2:**
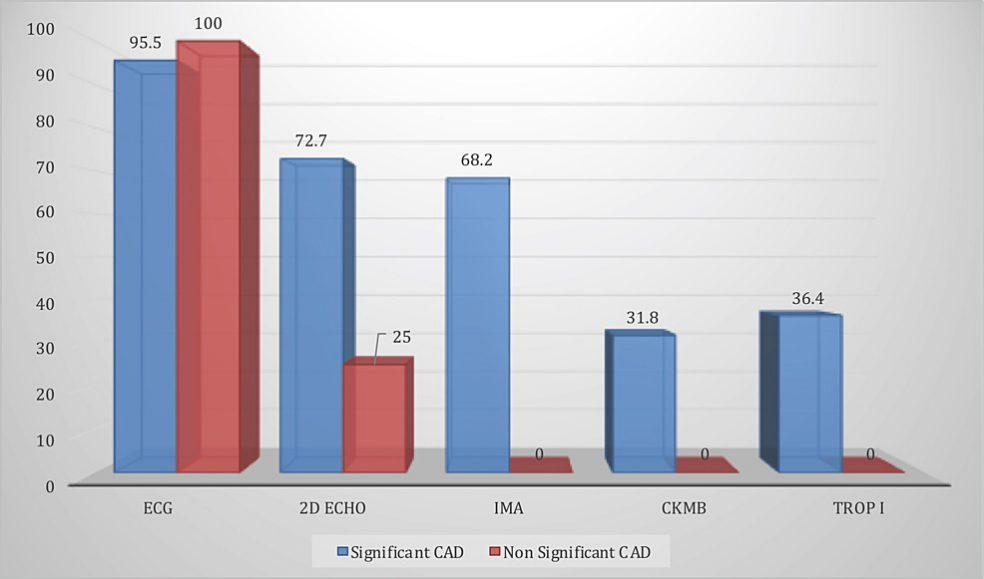
Diagnostic test findings in relation to significant CAD among cases (n=44) ECG: Electrocardiogram, 2D ECHO: 2D echocardiography, IMA: ischemia-modified albumin, CKMB: creatine kinase MB, TROP I: troponin I, CAD: coronary artery disease

Figures 1, 2 show the diagnostic test findings in relation to chest pain among cases. Among cases, who had typical chest pain (n=38), 47.4% had NSTEMI, 52.6% had STEMI, 71.7% had RMWA+ on 2D-Echo, 63.2% had IMA positive, 28.9% had CK-MB positive, 28.9% had Trop I positive, and 92.1% had significant CAD.

Table 4 shows the sensitivity and specificity of diagnostic tests in relation to CAG findings. 

**Table 4:** Sensitivity and specificity of diagnostic tests with relation to CAG findings Data presented as a percentage. TP: True positive, FP: false positive; FN: false negative; TN: true negative; PPV: positive predictive value; NPV: negative predictive value; IMA: ischemia-modified albumin; CK-MB: creatinine kinase-MB; ECG: electrocardiography

Test	TP	FP	FN	TN	Diagnostic Accuracy	Sensitivity	Specificity	PPV	NPV
ECG	43	12	7	38	81%	86%	76%	78%	84%
2D Echocardiography	35	4	15	46	81%	70%	92%	90%	75%
IMA	32	0	18	50	82%	64%	100%	100%	74%
CK-MB	15	0	35	50	65%	30%	100%	100%	59%
Troponin I	18	0	32	50	68%	36%	100%	100%	61%
ECG+IMA (any one +)	45	12	5	38	83%	90%	76%	79%	88%
2D Echocardiography+IMA (any one +)	39	4	11	46	85%	78%	92%	91%	81%
ECG+2D Echocardiography+IMA (any one +)	45	12	5	38	83%	90%	76%	79%	88%

A combination of ECG+IMA and ECG+IMA+2D-ECHO has the highest sensitivity (90 %) with 79% positive predictive value in the diagnosis of ACS within three hours of the onset of chest pain. However, ECG is equally sensitive (86% sensitivity, 78% positive predictive value). IMA alone has 64% sensitivity with 82% diagnostic accuracy and 100% positive predictive value. Among the biomarkers used, the diagnostic accuracy of IMA was the highest and better than troponin I and CK-MB. 

## Discussion

We studied 100 subjects (50 cases), presenting in the ED with chest pain typically indicative of angina pectoris or angina-like chest pain (non-cardiac chest pain). Patients who presented within three hours of the onset of chest pain were included in the study. Controls with non-cardiac causes of chest pain were included in the study for comparative analysis. 

In this study, cases were older as compared to controls and the majority, 34% were between 51 and 60 years of age. In the study conducted by Sahin et al., patients were between the ages of 62 and 79 [6]. The majority of the cases were males in the current study. In both groups, there were an equal number of male (33 each) and female (17 each) participants. Sarma et al. in their study also reported higher proportions of males [7]. 

Among cases, the majority (76%) had typical chest pain, and 24% had atypical chest pain. Among controls, only 28% had typical chest pain. It was discovered that atypical presentations occur greater often in individuals with non-ST-elevations ACS than in those with ST-elevation myocardial infarctions. Both women and men of advanced age often have atypical symptoms. Eighty-three percent of the patients in the research by El-Menyar et al. had conventional chest pains, whereas just 6% had atypical chest pains. Patients who presented with typical, atypical chest pains and dyspnea had death rates of 3%, 2.5% and 6%, respectively [8]. In the current study, 14% of cases and 10% of controls had CAD without overt heart disease. According to research by Wahrenberg et al., 4.7% of the 28,188 individuals developed ACS. In all, 8.2% and 32.4% of participants had a family history of early and ever-occurring CAD, respectively [9]. Jena et al. reported in the study of 50 cases of acute ischemic stroke, equal percentages of cases had diabetes and hypertension but a higher percentage of diabetics in the control group [10]. 

In the current study, only 4% of cases had normal ECG, 44% had NSTEMI, and 52% had STEMI on ECG. Several investigations have utilised wider criteria for ACS than the WHO criterion for myocardial infarctions and have chosen individuals having normal or non-diagnostic ECGs [11]. The results demonstrate that there were fewer diagnostically helpful clinical signs in individuals having a normal or non-diagnostic ECG, no significant comorbidity, and no evident alternative source for these symptoms [12]. 

In our study, out of 50 cases who underwent IMA, CK-MB and Troponin I tests, 32 (64%) had IMA positive,15 (30%) had CK-MB positive, and 18 (36%) had Trop I positive results. In a study by Collinson et al., among subjects (n=538), IMA or cardiac troponin was found to be raised in the admission sample of all patients with a final diagnosis of acute myocardial infarction (AMI) (n = 37). IMA alone was elevated in 2/37, Troponin alone in 19/37, and both were elevated in 16/37 subjects. In 173/501 patients in whom AMI was excluded both tests were found to be negative and in the non-AMI group, 22 patients had elevated IMA and cardiac troponin, both in the initial sample, suggesting AMI [13].

Out of 50 cases, 48 underwent coronary angiography in the present study. The majority 60.4% had double vessel disease (DVD), among whom, LAD+LCX was the most frequent (22.9%) affected vessels. This is in contrast with the studies conducted by Swain et al. and Dave et al., where authors have reported CAD patients’ high prevalence of single vessel disease (SVD) (68.7%) and triple vessel disease (TVD) (39.6%) respectively in their studies [14,15]. Swain et al. concluded that in young patients of CAD, SVD was predominantly in the STEMI group and TVD was predominantly seen in the NSTEMI/UA group.

Out of 50 controls in our study, the majority (n=30) had GERD as a cause of chest pain. Similarly, in a small sample of non-cardiac chest pain patients, Stahl et al. found that 61.5% of the subjects had GERD-related symptoms [16]. 

Findings from this study are in line with the study conducted by Kountana et al. in 33 patients (mean age 59.8±10.8 years; 28 men). The research comprised of patients who presented to the ED with severe chest pains lasting three hours indicative of an ACS having normal or non-diagnostic electrocardiograms and CK-MB and troponin values within the normal range. The authors concluded that serum IMA levels seem to be a valuable tool for excluding unstable angina in individuals having acute chest pains who report to the emergency room. Furthermore, IMA was found to have a negative predictive value (NPV) equivalent to echocardiography [17].

The current study has reported the sensitivity of IMA to be 64%, with a specificity of 100%, which is comparable with other studies [18-22]. We also reported that a combination of ECG+IMA and ECG+IMA+2D-ECHO has the highest sensitivity in the diagnosis of ACS within three hours of the onset of chest pain. However, ECG is equally sensitive. 

Our study has the limitations of a small sample size and patient enrollment from a single tertiary care centre. Thus, further study with a larger sample size is required to generalise the study findings for the population at large. 

## Conclusions

Time is crucial in the management of ACS and subsequent fatality can be markedly improved with quick confirmation of ACS and treatment as per guidelines. Hence, cardiac biomarkers play an important role in early confirmation of ACS. Cardiac Troponin I and CK-MB at times are found unreliable as they don't rise within three hours of onset of symptoms and thus new biomarkers with higher sensitivity and specificity must be identified to help us in prompt and early diagnosis. As found in our study, among the biomarkers used, the diagnostic accuracy of IMA was the highest and better than cardiac Troponin I and CKMB. Although ECG is the preferred diagnostic tool for diagnosing acute coronary syndrome (STEMI, NSTEMI and unstable angina) in patients presenting within three hours of the onset of chest pain, a confirmation can be done with the help of novel cardiac biomarkers and investigations like serum IMA levels and further treatment can be initiated.

## Figures and Tables

**Figure 3:**
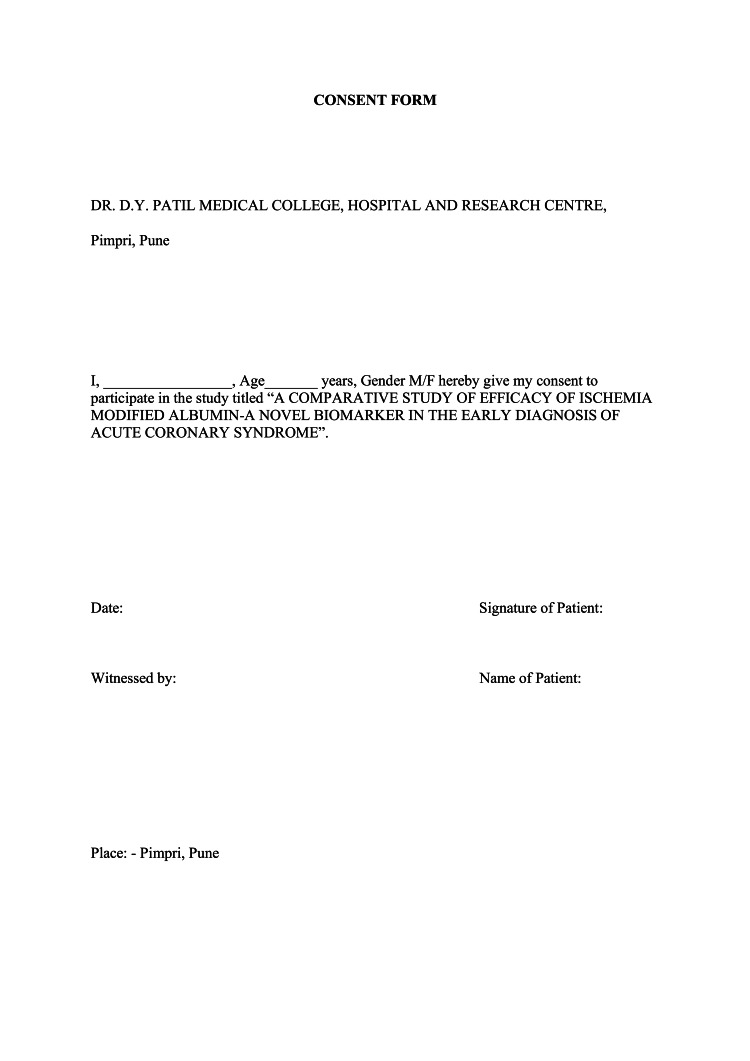
Consent form

## Appendices

**Table 5:** Pro forma CK-MB: CREATINE KINASE MB, HbA1c: GLYCOSYLATED HEMOGLOBIN A1c

DEMOGRAPHICS			
NAME			
AGE			
SEX			
ADDRESS			
OCCUPATION			
MOBILE NUMBER			
DATE OF ADMISSION			
DATE OF DISCHARGE			
IPD NUMBER			
PRN NUMBER			
CHIEF COMPLAINTS			
HISTORY OF PRESENTING ILLNESS			
MEDICAL HISTORY	YES	NO	
HYPERTENSION			
DIABETES MELLITUS			
THYROID DISORDER			
PERSONAL HISTORY	YES	NO	
SMOKING			
ALCOHOL			
TOBACCO			
DIET	YES	NO	
VEGITARIAN			
NON-VEGITARIAN			
GENERAL EXAMINATION			
TEMPERATURE			
PULSE			
BLOOD PRESSURE			
RESPIRATORY RATE			
SYSTEMIC EXAMINATION			
CARDIOVASCULAR EXAMINATION			
RESPIRATORY SYSTEM			
GASTROINTESTINAL SYSTEM			
CENTRAL NERVOUS SYSTEM			
INVESTIGATIONS			
ECG			
2D- ECHOCARDIOGRAPHY			
CK-MB			
TROPONIN I			
ISCHEMIA MODIFIED ALBUMIN			
CORONARY ANGIOGRAM			
TIMI SCORE			
GRACE SCORE			
BLOOD SUGAR LEVEL-FASTING			
BLOOD SUGAR LEVEL-POSTPRANDIAL			
HbA1c			
UREA			
CREATININE			
BILIRUBIN TOTAL			
BILIRUBIN CONJUGATED			
ALANINE TRANSAMINASE			
ASPARTATE TRANSAMINASE			
ALKALINE PHOSPHATASE			
TOTAL CHOLESTEROL			
TRIGLYCERIDES			
HIGH DENSITY LIPOPROTEIN			
VERY LOW DENSITY LIPOPROTEIN			
HIGH DENSITY LIPOPROTEIN			
